# Landslide disasters in eastern Uganda: post-traumatic stress disorder and its correlates among survivors in Bududa district

**DOI:** 10.1186/s40359-022-01001-5

**Published:** 2022-12-05

**Authors:** Amir Kabunga, Ponsiano Okalo, Viola Nalwoga, Brenda Apili

**Affiliations:** Department of Psychiatry, Lira University, P. O. Box 1035, Lira City, Uganda

**Keywords:** Mental health, Survivors of landslides, Post-traumatic stress disorder

## Abstract

**Background:**

Post-traumatic stress disorder is the commonly reported psychiatric morbidity among the survivors of natural disasters. However, its prevalence particularly in Bududa, Eastern Uganda is largely unknown.

**Aims and objectives:**

To assess the prevalence and correlates of post-traumatic stress disorder among Bududa landslide survivors.

**Methods:**

A community-based cross-sectional study was conducted on a sample of 587 participants. The study setting was the Bududa district, with a multistage sampling method. Pre-tested, administered interviewer PTSD Checklist–civilian version was used as screening tools between 4th and April 29th 2022. Data were analyzed using descriptive and binary logistic approaches at a 5% level of significance.

**Results:**

Of the study participants, 276 (46.8%) had PTSD symptoms. Among the respondents, 250 (42.6%) were in the age range of 40 and above, 396 (67.3%) were female, 284 (48.4%) had no formal education, and 333 (56.7%) were married. Results showed that male gender (AOR: 0.47; 95% CI 0.31–0.73; *p* = 0.001), widowhood (AOR: 0.44; 95% CI 0.21–0.94; *p* = 0.034), lack of counseling (AOR: 0.44; 95% CI 0.21–0.94; *p* = 0.001), and duration since the landslide (AOR: 0.35; 95% CI 0.23–0.52; *p* = 0.001) were associated with an increased likelihood of screening for PTSD in landslide survivors.

**Conclusion:**

Landslides pose significant effects on the mental health of survivors. In the present study, the extent of PTSD among survivors is substantially high. male gender,, widowhood, lack of counselling, low social support, and duration since the landslide were significantly associated with PTSD. Effective screening and awareness programs among survivors should be strengthened for the prevention and treatment of psychiatric morbidity among the survivors of landslides.

## Background

Natural tragedies including landslides, earthquakes, volcanic eruptions, hurricanes, floods and others kill close to 90,000 people affecting about 160 million others globally every year [1] Such disasters are traumatic and may result in a wide range of mental health problems [2] including depression, anxiety disorder, suicidal ideation and post-traumatic stress disorder (PTSD) are recorded [3]. Reports in low-income countries indicate that people are vulnerable in the aftermath of natural disasters [1]. The factors influencing peoples’ vulnerability include low levels of education, limited economic resources, poor infrastructure and poor health facilities [4]. Also, studies show that the impact of these events in terms of deaths, destruction, traumatic injuries, displacement of people and mental disorders recorded is much more severe in low and middle-income countries compared to high-income countries [5]. While physical problems tend to be addressed, many mental problems remain undetected [6].

PTSD is the most frequent, severe and debilitating reported psychiatric disorder reported in post-disaster [2]. It is characterized by intrusive memories, avoidance and persistent symptoms of increased arousal [7]. Studies have reported high levels of PTSD such as 37.3% among landslide survivors in Ethiopia [8], 36.3% among earthquake victims in Kerman [9], 75.5% among Rana Plaza building collapse in Bangladesh [10] and 57% in Saudi Arabia [11]. The occurrence and course of different mental disorders are affected by numerous risk factors [12]. These factors include gender, age, education levels, history of medical conditions, level of socioeconomic status, proximity to the disaster, level of exposure, the severity of injuries and social support [8]. These factors, however, vary from context to context. Thus, understanding these factors in the context creates the opportunity to develop context-based interventions to reduce and treat mental health disorders [13].

There is considerable heterogeneity in the distribution of exposure to traumatic events and some geographical areas are consistently exposed [14]. Bududa district is consistently exposed to large traumatic landslides and the overall exposure to trauma may exceed rates previously reported in other parts of the world. Over the past two decades, disastrous landslides have frequently hit the district. On 1st March 2010, the district experienced a major landslide of unprecedented magnitude burring three villages (Kubehwo, Namakansa and Nametsi) killing 400 people and leaving 5000 others displaced [15]. In 2012, a landslide left 450 people dead and many others displaced. In October 2018, 42 people were reportedly killed and more than 500 others were displaced. In December 2019, [6] people died in landslides following heavy rainfall, destroying several houses and crops. In 2020, more than 850 people were permanently displaced by landslides [16]. Between 2008 and 2020, more than 400 major landslides have been reported in Mount Elgon where Bududa district is located killing close to 1000 people [16]. Although PTSD is prevalent in post-disaster settings [8], many mental health problems after the disaster in low-income countries like Uganda go undetected leading to long-term psychiatric morbidity [17]. Therefore, the current study assessed the prevalence and correlates of PTSD among Bududa landslide survivors. This study may be important for early interventions or the reduction of PTSD burden among the victims.

## Methods

### Study setting and design

A community-based cross-sectional research design was employed to examine PTSD among Bududa landslide survivors in eastern Uganda. Data was collected in Bududa district which was the most affected district. Bududa district is situated in the surroundings of Mount Elgon volcano which is the hotspot for landslides in Uganda. Degradation of slopes through soil loss due to landslides in the district is a problem with fatalities [15].

### Study population

The study population included all adult landslide survivors in Bududa district in eastern Uganda. Survivors of landslides who were critically ill were excluded from the study.

### Sampling procedure

The sample size was 393 based on single population proportion estimation formula with assumptions of 37% prevalence of PTSD from a study in Addis Ababa, Ethiopia [8]. 0.37 p, 1.96 Z (standard normal distribution), 95% CI α =0.05 and a 10% nonresponse rate. Design effect 1.5 was considered and the total sample was 590. A multistage sampling technique was employed to select 590 participants affected by landslides. Cluster random sampling was used to select three villages of Kubehwo, Namakansa and Nametsi which were hotspots for landslides. A list of households of the survivors was obtained from District Disaster Preparedness and Management Committee. To select the households, a simple random sampling technique was used. Later the sample number was determined through proportionate allocation depending on the number of households in each village. Members of selected households were sorted for participation. In case of more than one eligible participant in a household, random selection was done through the lottery method. We went to the community asking for all adult survivors in the selected three villages. Only adults aged 18 years and above who had experienced landslides in Bududa district were included in the study.

### Data collection procedure

Data were collected using PTSD Checklist–civilian version (PCL-C-5), pre-tested administered interviewer questionnaire. PCL-C-5. PCL-C-5 consists of 17 items which comprise three domains, reexperiencing, avoidance, and hyperarousal 17. The three domains correspond to the DSM-IV symptoms for PTSD [18] on a 5 Likert scale based on the extent to which the respondent has been troubled by specific symptoms in the past month. The total possible score is calculated by summing up the scores for all the items and it ranges from 17 to 85 points. A total score of ≥ 50 indicates a full diagnosis [19]. In this study, the traumatic event in the original PCL-C-5 is replaced by landslides. In a variety of contexts, it has been discovered that PCL-C-5 has very good psychometric properties [20]. In a sample of Ethiopians, it demonstrated good internal consistency, dependability, and a strong link with PTSD diagnosis. It demonstrated a high-reliability score (Cronbach’s alpha = 096) [21]. In this study, Cronbach’s alpha for PCL-C was 0.84. The socio-demographic and landslide-related information questionnaire was developed by the researcher based on the literature review. Landslide-related variables included social support, counselling services, level of exposure, duration since the event, physical injury, history of mental health issues, loss of property and staying in camp. Data were collected by five trained community psychology students. These were trained on how to interview, ethical issues and obtaining participants’ informed consent. Written informed consent was obtained from the participants before data collection with information about the purpose and nature of the study. The participants were also informed that there was no foreseeable harm or risk as a result of the study. Data were between the 4th and April 29th 2022.

### Statistical analysis

The field tools were checked for completeness after the data collection process. The data was cleaned, coded, and exported into SPSS V-23 for analysis. For PCL-C-5, the final score was calculated by adding up each response item’s scores. Participants who received a score of > 50 were later identified as having PTSD [22]. In descriptive statistics, data were analyzed and summarized using frequencies, percentages, and measures of central tendency and dispersion. An association between PTSD and sociodemographic and landslide-related characteristics was examined using the Chi-Square test. The independent variable of PTSD was predicted using binary logistic regression analysis. With a *P*-value of 0.05 and a 95% confidence interval, the level of significance was set at 5%.

## Results

### Socio-demographic characteristics of the study participants (n = 587)

In Table 1, a total of 587 participants were included in the study, yielding a response rate of 99.4%. Of the study participants, 276 (46.8%) had PTSD symptoms. Among the respondents, 250 (42.6%) were in the age range of 40 and above years, 396 (67.3%) were female, 284 (48.4%) had no formal education and 333 (56.7%) were married. Results in Table 1 show that 129 (46.9%) of the participants aged 40 years and above, 215 (78.2%) who were married, 144 (52.4%) with no formal education and 142 (51.6%) who were married had PTSD.

**Table 1:** Socio-demographic characteristics associated with PTSD among study participants (n = 587)

Explanatory variables	Frequency (%)	Post-traumatic stress disorder	
Category		No ≤ 50 (n%)	Yes ≥ 50 (n%)
PTSD	587 (100)	312 (53.2)	276 (46.8)
* Age *			
18–28 years	126 (21.5)	76 (24.4)	50 (18.2)
29–39 years	211 (35.9)	115 (36.9)	96 (34.9)
≥ 40 years	250 (42.6)	121 (38.8)	129 (46.9)
* Gender *			
Male	192 (32.7)	132 (42.3)	60 (21.8)
Female	396 (67.3)	180 (57.7)	215 (78.2)
* Education level *			
No formal education	284 (48.4)	140 (44.9)	144 (52.4)
Primary education	207 (35.3)	113 (36.2)	94 (34.2)
Secondary education	70 (11.9)	38 (12.2)	32 (11.6)
Post-secondary education	26 (4.4)	21 (6.7)	5 (1.8)
* Marital status *			
Single	66 (11.2)	43 (13.8)	23 (8.4)
Married	333 (56.7)	191 (61.2)	142 (51.6)
Divorced/separated	96 (16.4)	44 (14.1)	52 (18.9)
Widowed	92 (15.7)	34 (10.9)	58 (21.1)

### PTSD landslide-related characteristics of the respondents

Results in Table 2 indicate that majority of the participants 176 (64.0%) who had direct exposure to the landslides, 208 (75.6%) who were affected within the duration of two years, 239 (86.9%) lost property due to landslides 254 (92.4%) who stayed in camps had elevated levels of PTSD.

**Table 2:** PTSD landslide-related characteristics of the respondents

Explanatory variables	Frequency (%)	PTSD	
Category		No ≤ 50 (n%)	Yes ≥ 50 (n%)
PTSD	587 (100)	312 (53.2)	276 (46.8)
*Level of exposure*			
Indirect	163 (27.8)	64 (20.5)	99 (36.0)
Direct	424 (72.2)	248(79.5)	176 (64.0)
*Duration since the event*			
After two years	216 (36.8)	149 (47.8)	67 (24.4)
Within two years	371 (63.2)	163 (52.2)	208 (75.6)
*Physical injury*			
No	410 (69.8)	233 (74.7)	177 (64.4)
Yes	177 (30.2)	79 (25.3)	98 (35.6)
*History of mental health issues*			
No	486 (82.8)	255 (81.7)	231 (84.0)
Yes	101 (17.2)	57 (18.3)	44 (16.0)
*Loss of property*			
No	99 (16.9)	63 (20.2)	36 (13.1)
Yes	488 (83.1)	249 (79.8)	239 (86.9)

### Factors associated with PTSD among landslide survivors

During the univariate logistic analysis (Table 3), the factors associated with PTSD among the study participants were being a female (COR: 0.38; 95% CI 0.26 − 0.055; *p* ≤ 0.001), being single (COR: 0.31; 95% CI 0.16–0.61; *p* ≤ 0.001), lack of counselling (COR: 3.41; 95% CI 2.39–4.83; *p* < 0.001), lack of social support (COR: 3.88; 95% CI 2.75–5.46; *p* < 0.001), indirectly exposed to landslide (COR: 0.45; 95% CI 0.31–0.65; *p* < 0.001), experienced landslides more than two years ago (COR: 0.35; 95% CI 0.25–0.50; *p* < 0.001), having physical injury (COR: 1.82; 95% CI 1.27–2.60; *p* = 0.007), loss of property (COR: 1.80; 95% CI 1.15–2.82; *p* = 0.022).

**Table 3:** Factors associated with PTSD among landslide survivors (n = 587)

Explanatory variables	Frequency (%)	PTSD		COR (95% CI)	*p*-value
Category		No ≤ 50 (n%)	Yes ≥ 50 (n%)		
PTSD	587 (100)				
*Age*					0.081
18–28 years	126 (21.5)	76 (24.4)	50 (18.2)	1.00	
29–39 years	211 (35.9)	115 (36.9)	96 (34.9)	0.68 (0.44–1.04)	
≥ 40 years	250 (42.6)	121 (38.8)	129 (46.9)	0.80 (0.56–1.16)	
*Gender*					< 0.001
Male	192 (32.7)	132 (42.3)	60 (21.8)	1.00	
Female	396 (67.3)	180 (57.7)	215 (78.2)	0.38 (0.26 − 0.055)	
*Education level*					0.020
No formal education	284 (48.4)	140 (44.9)	144 (52.4)	4.32(1.59–11.77)	
Primary education	207 (35.3)	113 (36.2)	94 (34.2)	3.49 (1.27–9.62)	
Secondary education	70 (11.9)	38 (12.2)	32 (11.6)	3.54 (1.21–10.44)	
Post-secondary education	26 (4.4)	21 (6.7)	5 (1.8)	1.00	
*Marital status*					< 0.001
Single	66 (11.2)	43 (13.8)	23 (8.4)	0.31 (0.16–0.61)	
Married	333 (56.7)	191 (61.2)	142 (51.6)	0.44 (0.27–0.70)	
Divorced/separated	96 (16.4)	44 (14.1)	52 (18.9)	0.69 (0.39–1.24)	
Widowed	92 (15.7)	34 (10.9)	58 (21.1)	1.00	
*Counselling*					< 0.001
No	213 (36.3)	73 (23.4)	140 (50.9)	3.41 (2.39–4.83)	
Yes	374 (63.7)	239 (76.6)	135 (49.1)	1.00	
*Social support*					< 0.001
No	270 (46.0)	96 (30.8)	174 (63.3)	3.88 (2.75–5.46) 1.00	
Yes	317 (54.0)	216 (69.2)	101 (36.7)		
*Level of exposure*					< 0.001
Indirect	163 (27.8)	64 (20.5)	99 (36.0)	0.45 (0.31–0.65)	
Direct	424 (72.2)	248 (79.5)	176 (64.0)	1.00	
*Duration since the event*					< 0.001
After two years	216 (36.8)	149 (47.8)	67 (24.4)	0.35 (0.25–0.50)	
Within two years	371 (63.2)	163 (52.2)	208 (75.6)	1.00	
*Physical injury*					0.007
No	410 (69.8)	233 (74.7)	177 (64.4)	1.82 (1.27–2.60)	
Yes	177 (30.2)	79 (25.3)	98 (35.6)	1.00	
*History of mental health issues*					0.467
No	486(82.8)	255 (81.7)	231 (84.0)	0.95 (0.63–1.47)	
Yes	101 (17.2)	57 (18.3)	44 (16.0)	1.00	
*Loss of property*					0.022
No	99 (16.9)	63 (20.2)	36 (13.1)	1.80 (1.15–2.82)	
Yes	488 (83.1)	249 (79.8)	239 (86.9)	1.00	

### A binary logistic regression analysis of factors associated with PTSD among landslide survivors

In multivariate analysis (Table 4), results showed that female gender (AOR: 0.47; 95% CI 0.31–0.73; *p* = 0.001), secondary education (AOR: 4.13; 95% CI 1.24–13.72; *p* = 0.021), widowhood (AOR: 0.44; 95 CI 0.21–0.94; *p* = 0.034), lack of counseling (AOR: 0.44; 95% CI 0.21–0.94; *p* = > 0.001), low social support (AOR: 2.27; 95% CI 1.52–3.38; *p* = > 0.001), experienced landslides more than two years ago (AOR: 0.35; 95% CI 0.23–0.52; *p* = > 0.001) and staying in camp (AOR: 0.50; 95% CI 0.27–0.94; *p* = 0.030) were associated with PTSD in landslide survivors.

**Table 4:** A Binary logistic regression Analysis of factors associated with PTSD among landslide survivors (n = 587)

Variables	COR 95% CI	AOR 95% CI	*p*-value
*Gender*			
Male	0.38 (0.26 − 0.055)	0.47 (0.31–0.73)	0.001*
Female	1.00		
*Marital status*			
Single	0.31 (0.16–0.61)	0.44 (0.21–0.94)	0.034*
Married	0.44 (0.27–0.70)	0.89 (0.50–1.58)	0.696
Divorced/separated	0.69 (0.39–1.24)	1.15 (0.59–2.25)	0.683
Widowed	1.00		
*Counselling*			
No	3.41 (2.39–4.83)	2.89 (1.90–4.40)	< 0.001*
Yes	1.00		
*Social support*			
No	3.88 (2.75–5.46)	2.27 (1.52–3.38)	< 0.001*
Yes	1.00		
*Duration since the event*			
After two years	0.35 (0.25–0.50)	0.35 (0.23–0.52)	< 0.001*
Within two years	1.00		

*Statistically significant variables at *p* < 0:05

## Discussion

The study of landslide survivors assessed the prevalence and correlates of PTSD symptoms in Bududa district, eastern Uganda. The primary finding showed that many years after devastating landslides, PTSD is prevalent among 46.8% (n = 276) of the landslide survivors and the factors independently associated with PTSD were female gender, secondary education, widowhood, lack of counselling, low social support, and duration since the landslide. These results tend to suggest that landslides had a strong influence on the mental health of landslide survivors with several contributing factors that need to be addressed.

The high level of PTSD recorded in our study may be attributed to the fact that the victims have been consistently exposed to large traumatic landslides. Over the past two decades, disastrous landslides have frequently hit the victims [16]. Also, the factors identified in our study including, the absence of counselling services and inadequate social support could have exacerbated this mental condition. Our finding confirms the results of a systematic review showing that the prevalence of PTSD among survivors ranged from 7.2 to 64% [23]. However, our results are higher than the 37.3% observed among the landslide survivors in Addis Ababa, Ethiopia [8], 36.3% among earthquake victims in Kerman [9] and 9.1% in southern Brazil [13]. The probable reason for this variation might be the difference in tools used, sample size and conducting studies late after the traumatic events. It is important to note, however, that years after the traumatic event, PTSD symptoms are experienced by landslide survivors and require specific diagnosis and intervention to prevent long-term psychiatric morbidity.

Our results showed significant socio-demographic risk factors for PTSD. In line with previous studies in Nigeria and Ethiopia among survivors of traumatic events [24, 25], the male sex was more likely to develop PTSD as compared to the female sex following landslides. However, contrally to our results, epidemiological surveys have shown that women are at least twice as likely as men to experience PTSD [26], attributing to the fact that females tend to experience child sexual abuse and sexual assault more than men [27]. Our results nonetheless, clearly show that the male survivors are also highly vulnerable, requiring special attention when providing post-disaster interventions. Regarding marital status, results showed that individuals who were single were less likely to develop PTSD compared to widowed participants. Marital status is the most widely researched factor concerning mental health conditions given its potential mediating role [28]. Although studies have provided mixed results [29], our findings are consistent with previous studies which have reported a high prevalence of PTSD among widowed participants [30]. Contrary to the current findings, studies show that people who are married tend to report low levels of mental health disorders compared to those who are unmarried [31].

Our results showed that landslide survivors who had not received counselling had close to three-fold increased odds of experiencing PTSD compared to those who had received counselling services. Given the severity of the harm caused by PTSD psychological options including counselling are offered. In line with the current findings, other studies have revealed that counselling was found to be significant in reducing PTSD [32, 33]. Similarly, female victims of trauma reported decreased PSTD after receiving counselling services [34]. Other studies have also demonstrated the effectiveness of counselling on PTSD [35].

Social support was significantly associated with PTSD in which those landslide survivors with poor social support were two times more likely to report PTSD compared to those with strong support. This implies that positive and adequate support mitigates the negative effects of the traumatic event. The presence of social support is considered one of the best protective factors against the development of PTSD [36]. Studies have demonstrated that social support available from different people including intimate partners, family members, and friends is significantly influential on better treatment outcomes for PTSD [37]. Conversely, studies have revealed that low levels of social support are associated with poor mental health outcomes [36]. In line with our findings, studies consistently demonstrate that inadequate social support is associated with more severe PTSD symptoms [38]. Also, our findings are consistent with the results of previous studies indicating that social support is associated with low or absence of PTSD following trauma exposure [39].

Duration since the traumatic event was found to be significantly associated with PTSD. Survivors who had experienced landslide events more than two years ago had 65% reduced odds of developing PTSD compared to those who had just experienced it within two years. The plausible explanation could be the gradual decline in PTSD over time. The results suggest that chronic forms of PTSD appear serious complications in the first few years of the occurrences of traumatic events. Thus, untreated PTSD especially in the first few months or years represents an extreme risk for mental health. This finding is supported by other studies in Ethiopia and South Africa [40]. Facing a disaster of this magnitude would be a different experience for the landslide survivors. These results are consistent with the findings of the study conducted in India among adolescents following floods [41].

### Strengths and limitations of the study

In this study, we used a standardized tool to assess the three clusters of PTSD symptoms severity based on DSM-1 V diagnostic criteria. Moreover, the tool has good psychometric measures. However, due to the cross-sectional nature of the study, the association between PTSD and different factors may not precisely imply causality. Also, few risk factors were included in the analysis, implying that only some possible results can be inferred. However, the sample is from a well-defined catchment area. Generally, there could have been certain biases, such as recall and social desirability.

## Conclusion

Landslides pose significant effects on the mental health of survivors. In the present study, the extent of PTSD among survivors is substantially high. male gender, widowhood, lack of counselling, low social support, and duration since the landslide were significantly associated with PTSD. Effective screening and awareness programs among survivors should be strengthened for the prevention of psychiatric morbidity among the survivors of landslides.

## Data Availability

All data analyzed during this study are available from the corresponding author on reasonable request.
